# Using Single Molecule mRNA Fluorescent *in Situ* Hybridization (RNA-FISH) to Quantify mRNAs in Individual Murine Oocytes and Embryos

**DOI:** 10.1038/s41598-018-26345-0

**Published:** 2018-05-21

**Authors:** Fang Xie, Kelsey A. Timme, Jennifer R. Wood

**Affiliations:** Department of Animal Science, University of Nebraska-Lincoln, 3940 Fair St, Lincoln, NE 68583-0908 USA

## Abstract

Changes in abundance of mRNAs during oocyte growth and maturation and during pre-implantation embryo development have been documented using quantitative real-time RT-PCR (qPCR), microarray analyses, and whole genome sequencing. However, these techniques require amplification of mRNAs, normalization using housekeeping genes, can be biased for abundant transcripts, and/or require large numbers of oocytes and embryos which can be difficult to acquire from mammalian species. We optimized a single molecule RNA fluorescence *in situ* hybridization (RNA-FISH) protocol, which amplifies fluorescence signal to detect candidate transcripts, for use with individual oocytes and embryos. Quantification using the software Localize showed patterns of *Gdf9* and *Pou5f1* mRNA expression in oocytes and embryos that were consistent with previously published data. Interestingly, low levels of *Nanog* mRNA were also accurately and reproducibly measured in oocytes and one- and two-cell embryos suggesting that RNA-FISH could be used to detect and quantify low abundance transcripts. Unlike other techniques, RNA-FISH is also able to detect changes in the localization patterns of mRNAs which may be used to monitor post-transcriptional regulation of a transcript. Thus, RNA-FISH represents an important technique to investigate potential mechanisms associated with the synthesis and stability of candidate mRNAs in mammalian oocytes and embryos.

## Introduction

Messenger RNAs are synthesized at a high rate in the oocyte. However, this transcriptionally active period is restricted to the oocyte growth phase with transcriptional quiescence coinciding with chromatin condensation prior to oocyte maturation and persisting through fertilization and the first cleavage stages of embryonic development^1,2^. In addition to high transcription rates during oocyte growth, the half-life of most oocyte mRNAs is long (~2 weeks) resulting in the accumulation of transcripts in the oocyte cytoplasm^3^. After fertilization, stored mRNAs undergo extensive post-transcriptional modifications which results in either protein translation and/or degradation until activation of transcription from the embryonic genome^4^. These initial characterizations were made based on global changes in mRNA synthesis and degradation using radiolabeling, 5-Bromouridine 5′-triphosphate (BrUTP) labeling, and non-specific stains (e.g. Hoechst). The use of microarray technologies demonstrated that mRNA degradation during oocyte meiotic maturation and after fertilization is selective with a subset of oocyte-expressed mRNAs retained in the developing embryo^5^. Likewise, microarray and RNA sequencing experiments have been used to monitor the dynamic changes in gene expression during pre-implantation embryonic development, which led to the identification of minor and major periods of zygotic genome activation^6–8^. Coupled with functional assays (e.g. knock-out mouse models), maternally expressed mRNAs that are essential for embryonic development (i.e. maternal effect genes) have also been identified^4,9^.

It is undeniable that these collective methodologies have produced a wealth of information about relative changes in the abundance of mRNAs during important periods of oocyte maturation and embryonic development. However, most of the data represents the average relative abundance of a transcript in a pool of cells that has often times been normalized to a constitutively expressed housekeeping transcript. Furthermore, these assays cannot identify changes in mRNA localization, which is an important component of post-transcriptional regulation of RNA storage, translation, and degradation including in maturing oocytes and pre-implantation embryos^10–13^. Finally, measuring mRNA abundance associated with increased transcription in growing oocytes, which are found in pre-antral follicles tightly associated with somatic granulosa cells^14^, can produce confounding results due to technical difficulties separating oocytes from the surrounding granulosa cells.

The development of single cell RNA sequencing has overcome some of these limitations, although it still requires linear amplification of cDNA prior to sequencing. It should be noted that this technique has been successfully used to identify changes in mRNA abundance in rhesus macaque, bovine, mouse, and human oocytes and/or embryos^15–19^. One-step RT-PCR assays have also been developed but a NCBI search showed that the majority of published data used this technique to primarily detect viral load in mammalian samples. Thus, there is a need for additional methods to reproducibly determine not only the absolute abundance of candidate mRNAs in individual cells but also changes in the location of these mRNAs in the oocyte or embryo during critical transitional periods in oocyte and embryo development (e.g. chromatin condensation, fertilization, and the maternal zygotic transition). In the current study, we modified a commercially available single molecule fluorescence *in situ* hybridization (RNA-FISH) technique which had previously been used to quantify and localize β-actin mRNA in neurons^20^ and human papillomavirus DNA in cervical cancer cell lines^21^. To determine the accuracy and validity of this method in oocytes and embryos, we analyzed the absolute abundance and localization of three well described transcripts, (*Gdf9*, *Pou5f1*, and *Nanog)* in mouse oocytes and embryos.

## Results

### Optimization of single molecule, branched DNA fluorescence *in situ* hybridization technique to detect the housekeeping transcripts Ppib, Polr2a and Ubc in individual murine oocytes

**Figure 1:**
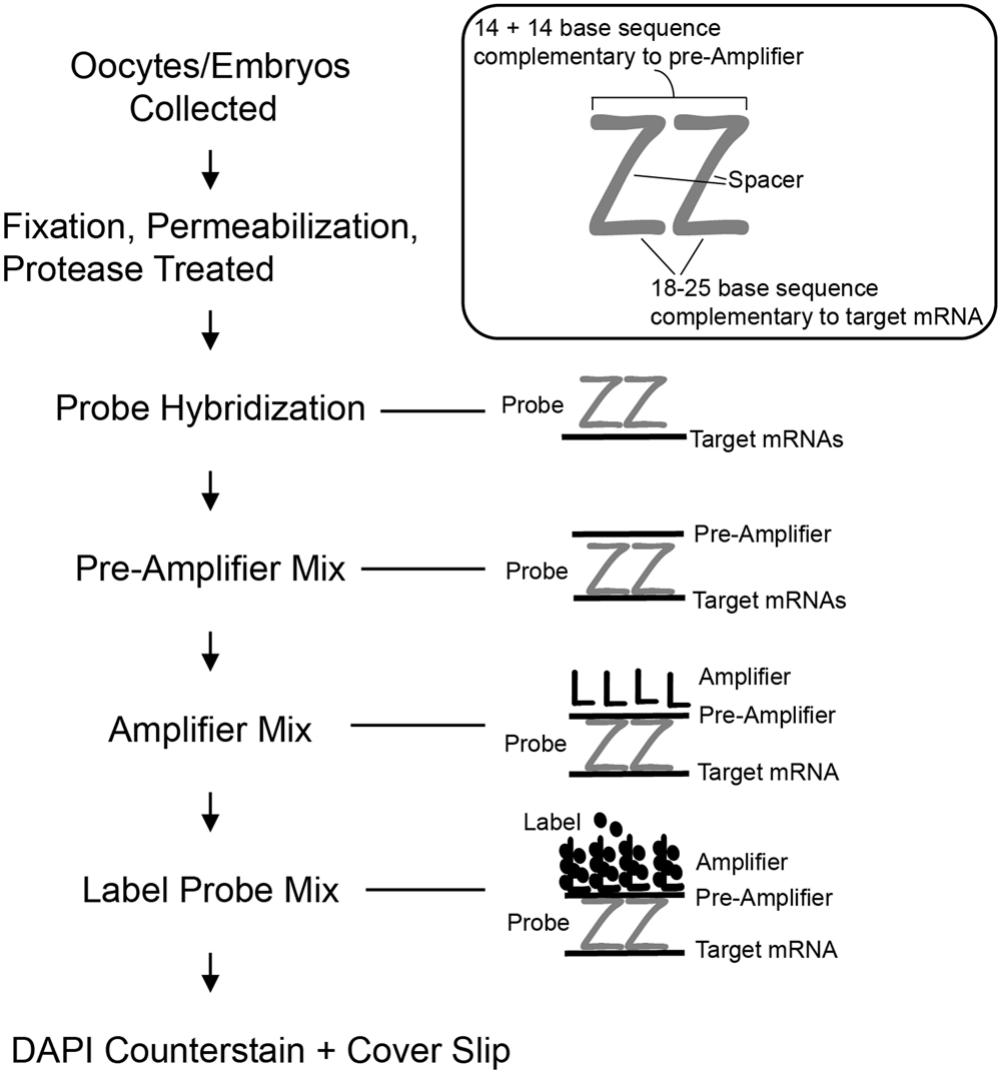
Single Molecule RNA-FISH Hybridization Scheme. Candidate mRNAs were detected by sequential hybridization with oligonucleotide probe pairs, pre-amplifier and amplifier probes, and fluorophore (label probe) which resulted in amplification of fluorescence signal. One side of each oligonucleotide probe pair was composed of sequence complementary to a specific target mRNA; that is, *Ppib*, *Ubc*, *Polr2a*, *Gdf9*, *Pou5f1*, *Nanog*, or *DapB* (inset box). The other side of the oligonucleotide pair contained complementary sequence to the pre-amplifier. Note that only 1 oligonucleotide pair is shown for simplicity; however, the oligonucleotide probes used were composed of 10–20 pairs that spanned the length of the target mRNA.

The objective of the first set of experiments was to optimize a single molecule RNA fluorescence *in situ* hybridization (RNA-FISH) protocol for use with oocytes and embryos. Commercially available assay kits were purchased from Affymetrix (QuantiGene ViewRNA ISH cell assay) and Advanced Cell Diagnostics (ACD, RNAscope Fluorescent Multiplex Assay). Both of these kits were designed using a similar chemistry; i.e., branched DNA technology, which amplifies the fluorescence signal rather than RNA or cDNA^21,22^. Specific to this experiment, proprietary probes for common housekeeping mRNAs including ubiquitin C (*Ubc*), peptidylprolyl isomerase B (*Ppib*), and RNA polymerase II subunit A (*Polr2a*) were purchased from ACD (Table 1). Likewise, a proprietary negative control probe which recognizes the *Bacillus subtilis* dihydrodipicolinate reductase (*DapB*) mRNA was also purchased (Table 1). Each of these probes were designed based on published NCBI sequence data for the murine or *B*. *subtilis* RNAs, respectively using an algorithm described by Bushnell *et al*.^23^ to optimize specificity. Each probe consisted of 10–20 oligonucleotide pairs with each of these pairs having a double Z configuration. One side of the Z (40–50 bases per pair) was complementary to a specific candidate transcript (e.g. *Ubc*) and the other side of the Z (14 + 14 bases) was complementary to pre-amplifier DNA sequence (Fig. 1). Sequential hybridization of pre-amplifier and amplifier molecules to the transcript-specific probes formed a branched DNA configuration which was subsequently bound by fluorophores (Fig. 1). This assembly structure has ~400 binding sites for each fluorophore, which generates an ~8000-fold amplification of the signal for each target RNA^24^. Together, this design effectively detected single mRNAs using standard fluorescence microscopy. The design also ensured specificity due to significant loss of fluorescence signal if both oligonucleotide Z pairs are not sequentially bound to the target RNA^22^. Likewise, at least 3 oligonucleotide pairs must be specifically bound to detect the fluorophores via standard microscopy imaging which also minimizes false positive detection of off target mRNAs.

**Table 1:** Probe Information for RNA-FISH.

Gene Name	Accession Number	Company	Catalog #	Probeset Target
*Gdf9*	NM_008110	Affymetrix	VB1-10331	bp 182–1280
*Pou5f1*	NM_013633	Affymetrix	VB6-14382	bp 222–1301
*Nanog*	NM_028016	Affymetrix	VB4-13553	bp 189–1187
*Ubc*	NM_019639	ACD	310771	bp 34–860
*Ppib*	NM_011149	ACD	313911	bp 98–856
*Polr2a*	NM_009089	ACD	312471	bp 2802–3678
*DapB*	EF191515	ACD	310043	bp 414–862

The exact sequences of the Quantigene (Affymetrix) and RNAScope (ACD) probesets are proprietary. The algorithm used to design the probes is described by Bushnell *et al*.^23^.

The assays kits from both Affymetrix and ACD were designed for tissues sections or adherent cells on microscope slides. Thus, in order to apply this technology to oocytes and embryos, several key adjustments were made. First, oocytes and embryos would not adhere sufficiently to cover slips even when coated with Poly-L-lysine solution. Instead, cells were placed into drops of 4% paraformaldehyde for fixation (see Materials and Methods) and were passed through drops of wash and hybridization buffers throughout the assay. Second, processing of oocytes and embryos with the proprietary permeablization and wash buffers supplied in the kit was not possible due to lysis of the oocytes and embryos. Therefore, we replaced these buffers with PBS-based permeablization and wash buffers that have previously been used for oocyte and pre-implantation embryo immunofluorescence experiments^25^. The permeabilization, wash, and hybridization steps were performed in Agtech 6-well plates (see Materials and Methods) with oocytes and embryos moved from well to well in order to complete these steps. For hybridization of transcript-specific probe, pre-amplifier DNA, and amplifier DNA, proprietary buffers supplied in either the QuantiGene ViewRNA Cell Assay kit (Affymetrix) or the RNAScope kit (ACD) were used. The oocytes and embryos were very fragile in this step, tended to float in the buffer, and became essentially transparent which initially made it difficult to find them post-hybridization. The use of the Agtech 6-well plates aided in locating the oocytes and embryos after hybridization because of the reduced surface area at the bottom of the well. Furthermore, it was important to alter the plane of focus when looking for the transparent cells as they were often submerged in the buffer but not fully settled onto the surface of the well. We attempted to use PBS-based buffers described for immunofluorescence for the transcript-specific probe, pre-amplifier DNA and amplifier DNA hybridizations. However, the end result was detection of fluorescence ringed around the plasma membrane of the oocyte suggesting aggregation of pre-amplifier DNA, and/or amplifier DNA which prevented their entry into the oocyte. Therefore, care was taken to make sure that oocytes and embryos were submerged in the propriety hybridization solutions throughout the incubation periods and cells were gently moved from hybridization to the PBS-based wash buffers in order to minimize lysis.

**Figure 2:**
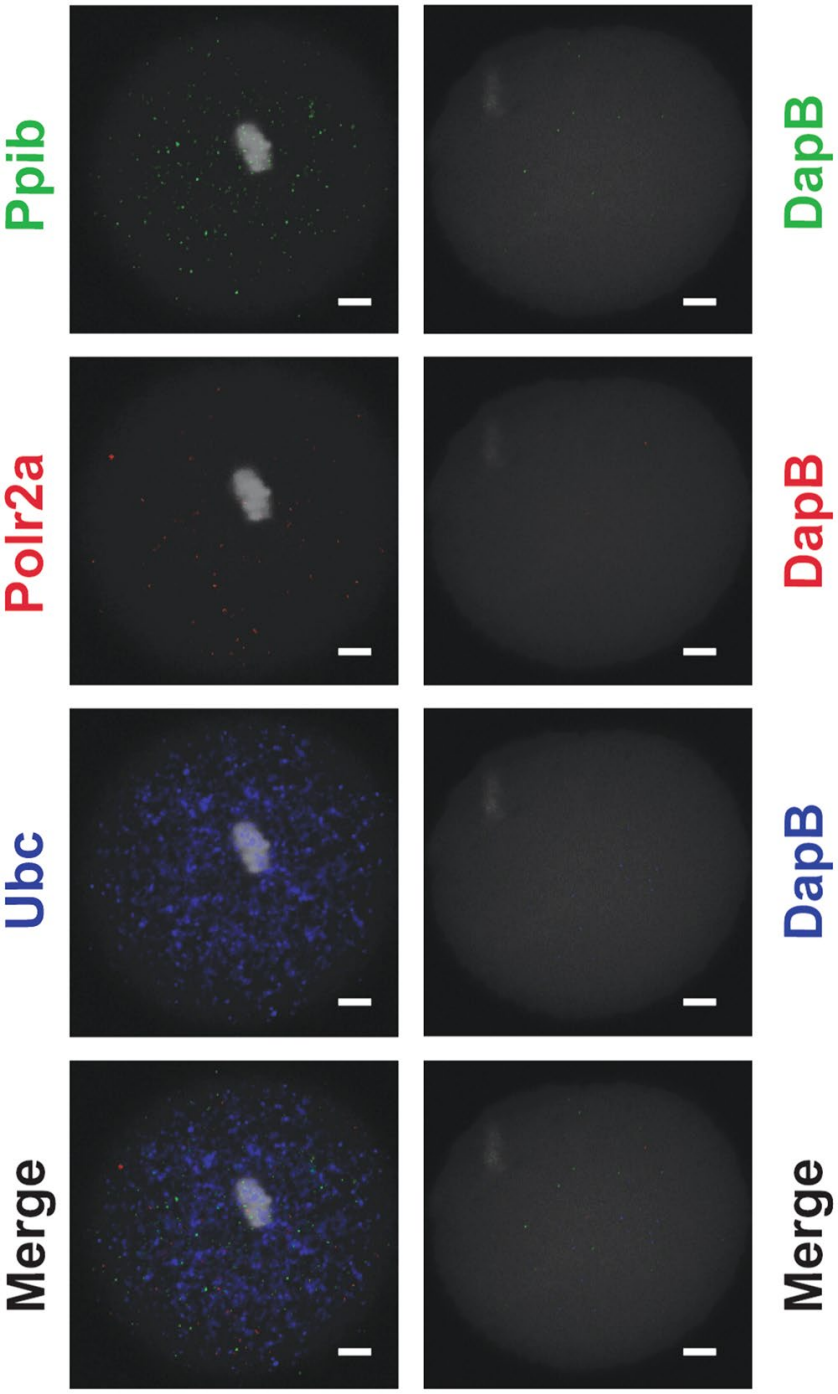
Representative Confocal Microscopy Images of RNA-FISH Performed with *M*. *musculus* Positive Control and *B*. *subtilis* Negative Control Probe Sets. Transcripts for *Ppib* (488 nm, green), *Polr2a* (550 nm, red), and *Ubc* (647 nm, blue) were detected as punctate spots in individual *in vivo* matured oocytes (MII oocyte). Conversely, there was a lack of punctate spots detected for each fluorophore when MII-oocytes were hybridized with *DapB*-specific probe set. The image for each transcript in each oocyte represented the middle z-section generated by confocal microscopy. All cells were counterstained with DAPI (greyscale in each image). The white scale bar for each image is 10 μm.

Using this protocol, individual, MII oocytes were hybridized with probes specific for *M*. *musculus Ubc*, *Ppib*, and *Polr2a* probes using a multiplex strategy. Alternatively, MII oocytes were hybridized with probe specific for *B*. *subtilis DapB* which is not expressed in mammalian cells. After hybridization with each of the 4 transcript-specific probes, pre-amplifier and amplifier hybridization was performed followed by application of fluorophores specific to each probe (*Ubc* = 647 nm, *Ppib* = 488 nm, and *Polr2a* = 550 nm). It should be noted that *DapB* was hybridized with each of the 3 fluorophores (647 nm, 448 nm, and 550 nm) and therefore represented a negative control for *Ubc*, *Ppib*, and *Polr2a*. Images from confocal microscopy showed punctate signal for each of the mammalian housekeeping mRNAs (Fig. 2). As expected the fluorescence intensity was visually highest for *Ubc* (high expresser), intermediate for *Ppib* (moderate expresser), and lowest for *Polr2a* (low expresser). Furthermore, there was no overlapping signal for each RNA (Fig. 2, merged image) and imaging of the MII oocytes hybridized with *DapB* in each fluorophore channel showed little to no signal (Fig. 2). These data as well as the algorithm used to design transcript specific probes described above demonstrated specificity of this assay.

### Quantification of individual mRNA molecules for Ubc, Ppib, Polr2a in MII-oocytes and 2-cell embryos using Localize

Each punctate fluorescence signal in each confocal image was representative of a single mRNA molecule and therefore data from these images could be quantified. Although confocal microscopy produces high resolution images, manual counting of the signal proved difficult due to the large size of the oocyte and the inability to objectively exclude spots with low fluorescence intensity which could be attributed to background noise. To overcome these obstacles, we used the software program Localize, which is a fluorescent particle identification and counting program written in Interactive Data Language (IDL). The program identifies the center of each signal detected by microscopy while excluding background signal (i.e. noise) based on a photon threshold determined by a Gaussian mask fitting algorithm^26^. In this way, the program counts any signal that exceeds this threshold which in our case was an individual mRNA; signal below the threshold were not counted.

**Figure 3:**
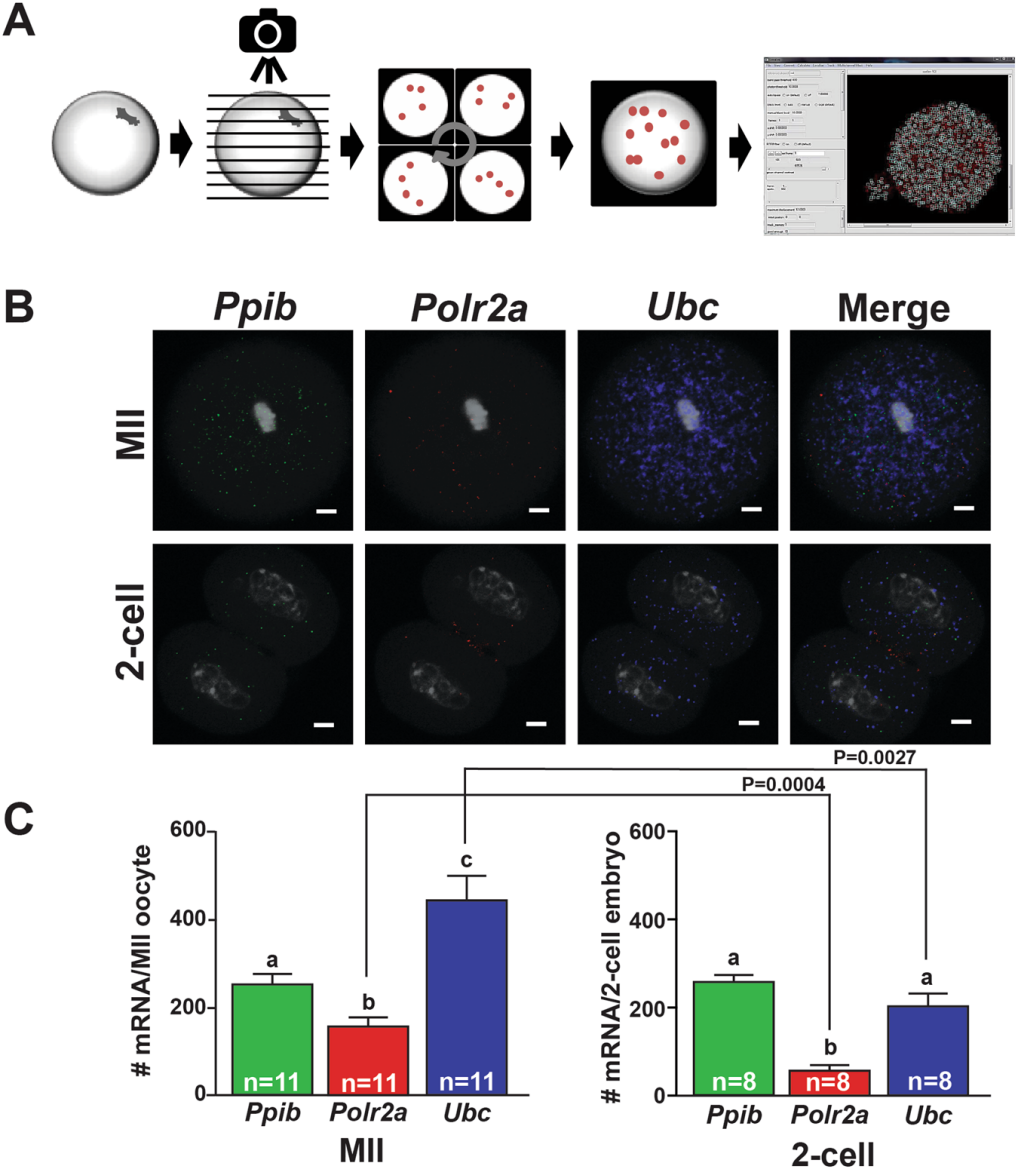
Quantification of Ppib, Polr2a, and Ubc mRNAs in MII-oocytes and 2-cell embryos using Localize. (**A**) Flow chart depicting image collection by conofocal microscopy, generation of a composite image by stitching together individual z-sections in Image J-Fuji, and output when images were analyzed using Localize. The default band pass and photon thresholds used for these analyses were 400 and 10.0000 respectively. (**B**) Representative images (middle z-section) of MII-oocytes and 2-cell embryos hybridized with probe sets specific to *Ppib*, *Polr2a*, and *Ubc*. These three probe-sets were multi-plexed and a representative merged image (Merge) is also shown. The white scale bar for each image is 10 μm. (**C**) The average number of *Ppib* (green bars), *Polr2a* (red bars), and *Ubc* (blue bars) transcripts (±SEM) detected by RNA-FISH and counted by Localize in individual MII-oocytes and and 2-cell embryos. The number (n) of oocytes and embryos analyzed for each transcript is indicated in each bar. One-way ANOVA and Tukey pair-wise comparison post-test analyses were performed to compare the abundance of each mRNA in each oocyte and embryo stage. Statistical significance was defined as P < 0.05 and is indicated by differences in letters above the bars. Significant differences in transcript abundance between developmental stages was determined by Student’s t-test.

In order to count fluorescence particles, Z-series images with maximum projection were obtained from confocal microscopy of MII-oocytes or 2-cell embryos that were subjected to RNA-FISH using the transcript-specific probes for *Ppib*, *Polr2a*, *Ubc*, or *DapB*. Sequential Z-images were subsequently stitched together using the Grid/Collection stitching plug-in for Image J Fiji (Fig. 3A). The plug-in, which was designed by Preibisch *et al*.^27^, determines the best overlap of fluorescence signal from the sequential images and uses this information to optimally construct a composite image. In our experiment, it enabled us to produce a composite image for each oocyte or embryo that could be used to count individual fluorescence particles using Localize without over counting particles from individual Z-series images. To count the fluorescence associated with each transcript specific probe, the Localize program was run with the output being the number of spots counted (Fig. 3A). The analysis was performed using images generated from MII-oocytes (n = 11) and 2-cell embryos (n = 8) hybridized with probes for *Ppib*, *Polr2a*, and *Ubc* (Fig. 3B). The number of transcripts for *Ppib* (255.4 ± 21.9), *Polr2a* (158.5 ± 19.07), and *Ubc* (445.1 ± 55.61), were counted in MII oocytes (n = 11, Fig. 3C). Transcripts for *Ppib* (253.4 ± 17.11), *Polr2a* (50.88 ± 11.31), and *Ubc* (197.4 ± 30.62) were similarly identified and counted in 2-cell embryos by Localize (Fig. 3C). These average transcript numbers for each housekeeping gene showed low variability between cells within a developmental stage and statistical analysis indicated significant differences (P < 0.0095) between *Ppib*, *Polr2a*, and *Ubc* transcript numbers consistent with predicted expression levels. Furthermore, we identified significant differences in the mRNA abundance of *Polr2a* (P < 0.004) and *Ubc* (P < 0.0027) between oocytes and embryos (Fig. 3C) which is consistent with both anecdotal information and a comprehensive study performed by Mamo *et al*.^28^. Image stitching and Localize analysis of MII-oocytes (n = 4) hybridized with *DapB* and each fluorophore showed high variability and low counts (18 ± 10.1, 488 nm; 18.7 ± 9.4, 550 nm; and 91 ± 28.6, 647 nm).

### Expression profile of Gdf9, Pou5f1, and Nanog in cumulus-oocyte complexes (COCs), MII oocytes, 1-cell embryos, and 2-cell embryos

**Figure 4:**
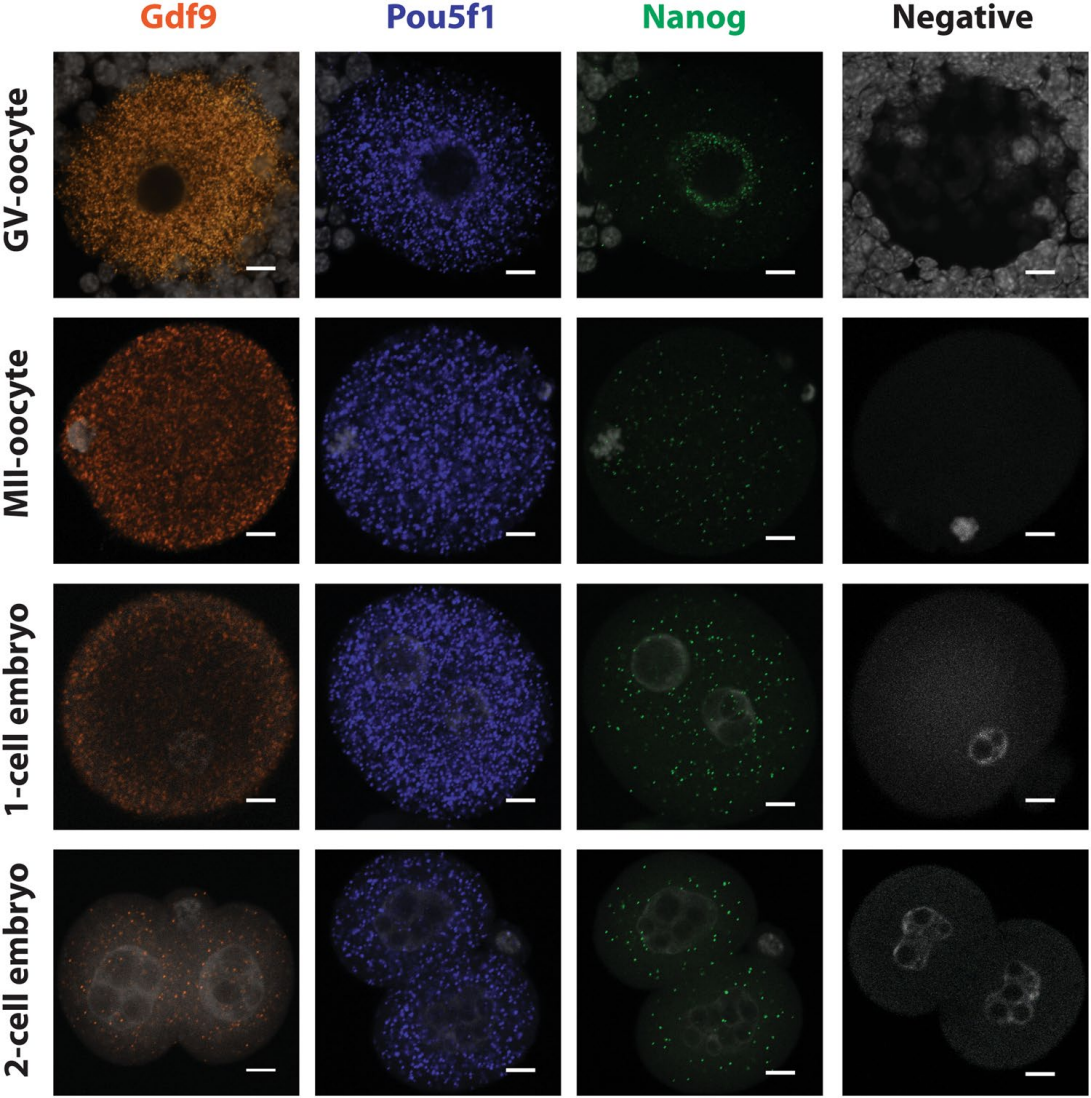
Representative Confocal Microscopy Images of *Gdf9*, *Pou5f*1, and *Nanog* mRNAs in oocytes and embryos. Transcripts for *Gdf9* (550 nm, orange), *Pou5f1* (650 nm, blue), and *Nanog* (488 nm, green) were detected as punctate spots in individual cumulus oocyte complexes (GV oocyte), *in vivo* matured oocytes (MII oocyte), 1-cell embryos and 2-cell embryos. The image for each transcript in each oocyte or embryo represented the middle z-section generated by confocal microscopy. All cells were counterstained with DAPI (greyscale in each image). Only DAPI signal was detected in oocytes or embryos incubated with only the pre-amplifier, amplifier, and fluorophore (Negative). The white scale bar for each image is 10 μm.

The next objective was to examine and quantify the expression profile of known oocyte and/or embryo specific transcripts using RNA-FISH. Briefly, cumulus oocyte complexes (COCs) containing germinal vesicle (GV) oocytes (n = 42), presumptive MII oocytes (n = 36), 1-cell embryos (n = 28), and 2-cell embryos (n = 20) were each collected from at least 4 CD-1 female mice that were stimulated with eCG or eCG/hCG (see Materials and Methods). RNA-FISH was subsequently performed using transcript specific probes for *Gdf9*, *Pouf51*, or *Nanog* (Table 1) as described for the housekeeping transcripts. Hybridized mRNAs for *Gdf9*, *Pou5f1*, and *Nanog* were labeled with 550 nm, 647 nm, and 488 nm fluorophores, respectively and detected using confocal microscopy. As expected, robust staining for *Gdf9* and *Pou5f1* mRNAs was detected in the GV-oocytes (Fig. 4). Unexpectedly, specific staining for *Nanog* was also detected in GV-oocytes with the majority of transcripts overlaying the nucleolus region (Fig. 4). Transcripts for *Gdf9* continued to be detected in MII-oocytes, 1-cell embryos, and 2-cell embryos. However, signal strength was diminished at each stage. Interestingly, the *Gdf9* transcripts seemed to preferentially localize to the subcortical region of the 1-cell embryo (Fig. 4). Signal strength of *Pou5f1* was maintained at each oocyte and embryo stage which is consistent with its classification as a maternal effect gene; i.e., it would be retained during oocyte maturation and early embryonic development (Fig. 4). Likewise, *Nanog* transcripts also continued to be detected in MII-oocytes as well as 1-cell and 2-cell embryos with even distribution of these transcripts in the cytoplasm (Fig. 4). Specific fluorescence detection of *Gdf9*, *Pou5f1*, and *Nanog* was verified by omission of any transcript specific probe in the negative control cells (Fig. 4) and the low-level detection of *DapB* in MII-oocytes (Fig. 2). Lack of *Gdf9*, *Pou5f1*, or *Nanog* hybridization in the cumulus granulosa cells surrounding the GV-oocytes also was indicative of probe specificity.

### Quantification of Gdf9, Pou5f1, and Nanog in oocytes and embryos using Localize

**Figure 5:**
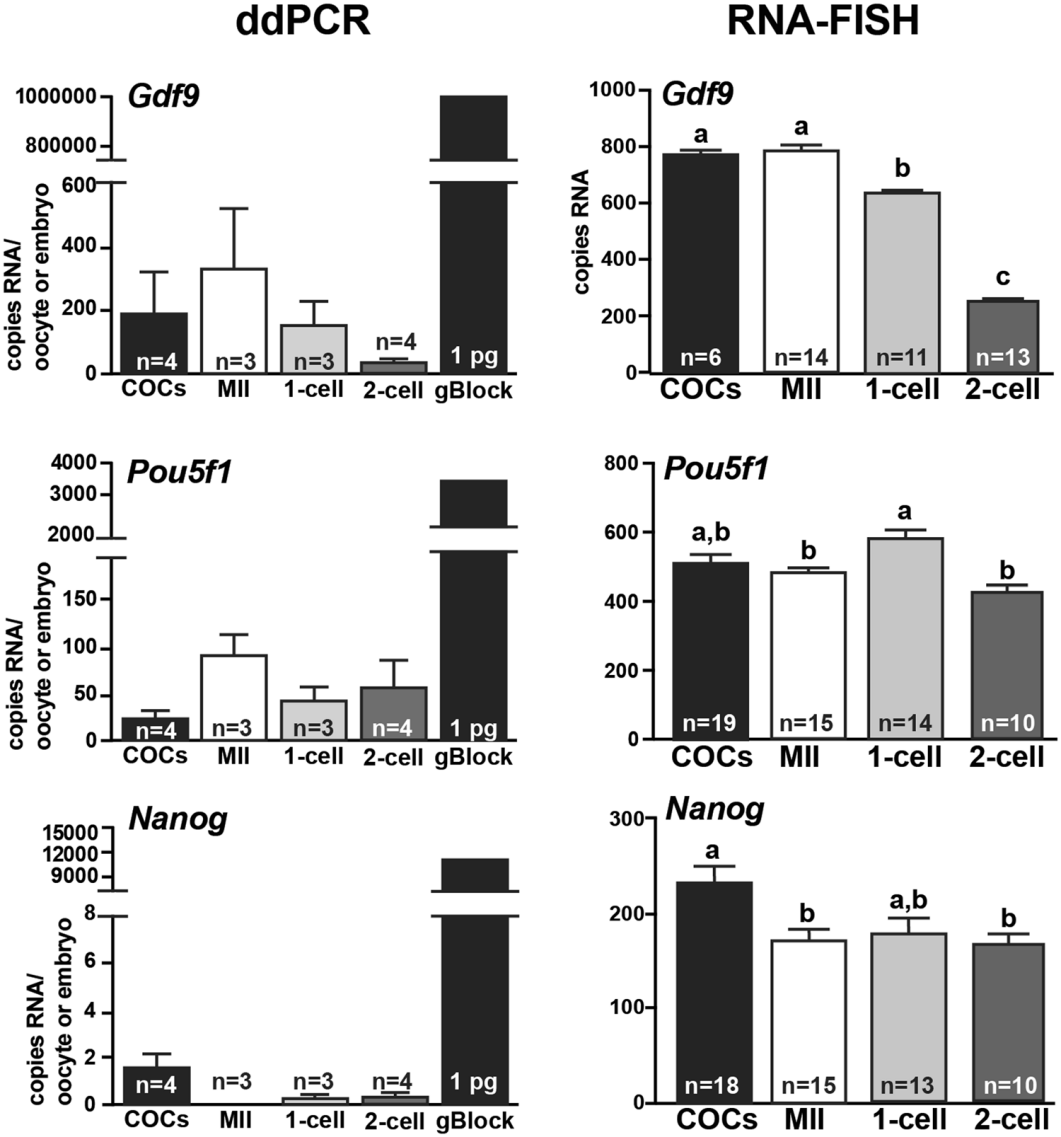
Copy numbers per oocyte or embryo of *Gdf9*, *Pou5f1*, and *Nanog* mRNAs were quantified by Localize analysis of RNA-FISH assays or droplet digital PCR (ddPCR) analysis of cDNA. (**A**) The average number of *Gdf9*, *Pou5f1*, and *Nanog* transcripts in cumulus oocyte complexes (COCs, black bars), MII-oocytes (white bars), 1-cell embryos (light grey bars) and 2-cell embryos (dark grey bars) was determined based on ddPCR (left side) or RNA-FISH (right side). Each ddPCR reaction was performed using 3–4 biological replicates of cDNA with each replicate generated from a pool of 15–20 oocytes or embryos. The number of each transcript in each oocyte or embryo (y-axis) was calculated as described in Methods. Standard error of the mean (SEM) within each experimental group is shown. One pg of gBlock synthetic DNA for each candidate gene served as the positive control for each ddPCR assay. (**B**) The average number of *Gdf9*, *Pou5f1*, and *Nano*g transcripts (±SEM) detected by RNA-FISH and counted by Localize in individual GV-oocytes (black bar), MII-oocytes (white bar), 1-cell embryos (light grey bar), and 2-cell embryos (dark grey bars) are shown. The number (n) of oocytes and embryos analyzed for each transcript is indicated in each bar. One-way ANOVA and Tukey pair-wise comparison post-test analyses were performed to compare the abundance of each mRNA in each oocyte and embryo stage. Statistical significance was defined as P < 0.05 and is indicated by differences in letters above the bars.

Confocal microscopy produced serial images which were stitched together using ImageJ-Fiji and counted using Localize as described for the housekeeping mRNAs. Transcripts encoding *Gdf9* were most abundant in GV-oocytes (777 ± 8 transcripts) and this number steadily declined to 646 ± 7.8 transcripts in 1-cell embryos and 262 ± 9.7 transcripts in 2-cell embryos (Fig. 5). Conversely, 423–576 *Pou5f1* transcripts were detected in GV-stage oocytes through 2-cell embryos with the highest number of Pou5f1 transcripts in 1-cell embryos (Fig. 5). Interestingly, 169–233 *Nanog* transcripts were consistently detected in oocytes and 1-cell and 2-cell embryos (Fig. 5).

### Droplet Digital PCR analysis of Gdf9, Pou5f1, and Nanog transcripts in MII oocytes and 1- and 2-cell embryos

Reverse-transcription polymerase chain reaction (RT-PCR) amplification of cDNA has been the gold standard for measuring the abundance of candidate transcripts within a cell. The development of quantitative PCR methods including real-time RT-PCR (qPCR) and droplet digital PCR (ddPCR) has improved the quantitative power of these techniques. Thus, we compared the absolute quantification of *Gdf9*, *Pou5f1*, and *Nanog* mRNAs determined by RNA-FISH to the abundance of each transcript using ddPCR. Briefly, RNA from GV-oocytes, presumptive MII-oocytes, 1-cell embryos, and 2-cell embryos was collected, reverse transcribed, and the resulting cDNA used in ddPCR reactions containing primers for *Gdf9*, *Pou5f1*, or *Nanog* (Table 2). Each biological replicate for ddPCR represented cDNA collected from 15–20 oocytes or embryos and 3–4 biological replicates were analyzed per each developmental group. Numerically, the abundance of *Gdf9* was highest in the MII oocytes (333.7± 187.4) and steadily declined in the 1-cell (151.3 ± 77.83) and 2-cell embryo (33.29 ± 15.26); however, there were no statistical differences in mRNA abundance between the oocytes and embryos due to high variation (Fig. 5). As expected, *Pou5f1* transcripts were numerically stable in the COCs (23.98 ± 10.49), MII oocytes (90.5 ± 21.35), 1-cell embryos (44.15 ± 13.77), and 2-cell embryos (57.33 ± 29.63) (Fig. 5). Finally, Nanog was detected in COCs (1.52 ± 0.65) but was inconsistently detected at the other developmental stages (Fig. 5). It should be noted that these transcript numbers are lower than what was observed by RNA-FISH; however, the relative expression profile for each transcript at each transcriptional stage was similar between the two techniques.

**Table 2:** PCR Primer Sequences and gBlocks Gene Fragment Sequences for ddPCR Experiments.

Gene Name	Accession Number	Forward Primer	Reverse Primer	gBlock
*Gdf9*	NM_008110	5′-GCC GGG CAA GTA CAG CC-3′	5′-TTT GTA AGC GAT GGA GCC G-3′	bp 121–1446
*Pou5f1*	NM_013633	5′-GAG GAG TCC CAG GAC ATG AAA G-3′	5′-GCT TCA GCA GCT TGG CAA AC-3′	bp 69–1127
*Nanog*	NM_028016	5′-AAG CGG TGG CAG AAA AAC C-3′	5′-GTG CTG AGC CCT TCT GAA TCA-3′	bp 219–1136

Primers and gBlock Gene Fragment Sequences were purchased from Integrated DNA Technologies. The gBlock base pairs (bp) indicate the region of the reference sequence that was used to design the gBlock.

## Discussion

*In situ* hybridization (ISH) for detection of DNA and RNA in histological sections was described by Pardue and Gall^29,30^ and John *et al*.^31^ in 1969. Over the years, modifications to ISH have replaced radiolabeling with fluorophore labeling as the method of detection. Furthermore, sensitivity of the assay has been improved. For example, coupling of PCR or RT-PCR amplification of target DNA or RNA, respectively with ISH has been used to detect low abundance transcripts, single copy genes, and viral and other foreign DNAs^32^. Despite the increased sensitivity of PCR coupled ISH, the technique is complex and dependent on PCR efficiency. Therefore, reproducibility of assays can be difficult to attain and, in some cases, frequent false negative or false positive results can occur. Furthermore, the data is only semi-quantitative unless the PCR and ISH are followed by flow cytometry^32^. To circumvent these problems, signal amplification chemistry has been improved in order to enable detection of single molecules of DNA or RNA. Examples of this improved signal detection includes the use of multiple short oligonucleotide probes conjugated with fluorescent dyes or attachment of an enzymatic reporter to the probe (e.g. HRP)^33,34^. Additionally, Player *et al*.^21^ described the development of branched DNA based probes for use in ISH as a sensitive method to detect human papillomavirus DNA in intact cells. This technology has also been used to detect both mRNAs and viral DNAs in adherent fixed cells as well as formaldehyde fixed paraffin embedded (FFPE) tissue sections^22,33^. Our study modified the RNA-FISH procedure to detect housekeeping (*Ubc*, *Polr2a*, and *Ppib*) mRNAs in MII-oocytes and 2-cell embryos while oocyte/embryo-specific (*Gdf9*, *Pou5f1*, and *Nanog*) mRNAs were detected in cumulus-oocyte complexes, MII oocytes, and pre-implantation embryos. Procedurally, the proprietary permeabilization and wash buffers supplied in the commercially available kits were replaced with PBS-based wash buffers to prevent cell lysis. Oocytes and embryos were also subjected to hybridization and wash steps in small volumes of reagents in AgTech 6-well plates and secured to slides in mounting media. These modifications along with the amplification of fluorescence afforded by the branched DNA chemistry allowed for detection of individual transcripts. The specificity of this detection was validated by the low to undetectable levels of fluorescence when cells were hybridized with transcript-specific probe designed against the *Bacillus subtilis DapB* mRNA and when the assay was performed omitting a transcript-specific probe.

Buxbaum *et al*.^20^ coupled the branched DNA ISH chemistry with the software program Localize^26^ in order to quantify the number of β-actin mRNAs in different regions of a neuronal dendrite. We used this same software analysis to count mRNAs for *Ubc*, *Polr2a*, *Ppib*, *Gdf9*, *Pou5f1*, and *Nanog* in oocytes and embryos. Confocal images were stitched together which generated a composite image that was subsequently counted (Fig. 3). The development of the stitching plug-in for ImageJ-Fiji^27^ ensures optimal overlapping of signal and thereby reduces overcounting of the same signal present in multiple Z-series images. While the sizes of individual mRNAs are generally smaller (~300 nm) than the thickness of each Z-series image, the extended conformation of the branched DNA upon which the fluorophores adhere likely results in a molecule with a much larger size. Taken together, we assume that counts were not inflated for each transcript due to the use of the stitching algorithm and we were likely not under-counting transcripts due to the extended size of the signal.

The data generated from the RNA-FISH analyses showed well-described profiles of *Gdf9* and *Pou5f1* mRNA abundance between immature oocytes, mature oocytes, and pre-implantation embryos consistent with previously reported data^6,35,36^. Reich *et al*.^37^ previously showed, using microarray analyses, that mRNAs expressed in the human MII-oocyte were also found in the associated polar body. Indeed, both *Gdf9* and *Pou5f1* transcripts were also localized in the polar body of MII-oocytes and 2-cell embryo using RNA-FISH (Fig. 4). Interestingly and unexpectedly, low but specific signal for *Nanog* was detected in GV and MII oocytes as well as 1- and 2-cell embryos (Figs 4 and 5). The levels of *Nanog* transcript in MII-oocytes (173.2 ± 11.55) were significantly higher than the detection of DapB (18 ± 10.1) in MII-oocytes using the same fluorophore (488 nm). Furthermore, no *Nanog* mRNA was visualized by RNA-FISH in the granulosa cells associated with GV-oocytes indicating specific detection of this transcript in oocytes and embryos (Fig. 4). Droplet digital PCR analysis also identified low abundance of Nanog mRNAs in oocytes and embryos; however this detection was inconsistent suggesting that transcript abundance is at the sensitivity level of this assay (Fig. 5). Synthesis of a full-length mRNA is dependent on transcriptional initiation and elongation. Interestingly, Guenther *et al*.^38^ showed that transcriptional initiation occurs at both “active” and “inactive” genes with the synthesis of detectable mRNAs regulated by productive versus non-productive elongation of the transcript. Given that the RNA-FISH technique described in this study can detect transcripts with the hybridization of only 3 oligonucleotide probe pairs, the detection of *Nanog* mRNAs in the oocyte and early pre-implantation embryo may be reflective of transcriptional initiation during oocyte growth which may or may not produce full-length mRNAs dependent on regulation of transcriptional elongation. However, the *Nanog* hybridization produced bright punctate signal and therefore, low-levels of full-length *Nanog* mRNAs may be synthesized in the oocyte which may or may not be translated into functional protein. Regardless, these data demonstrate the sensitivity of the RNA-FISH technique, which could be monopolized to more clearly define the regulation of mRNA synthesis and post-transcriptional stability in mammalian oocytes and embryos.

We propose that the RNA-FISH technique described in this manuscript represents an important tool for several lines of investigation in reproductive physiology and developmental biology. For example, it may be used to assess how localization patterns of mRNAs change during growth and maturation of oocytes and embryos in the absence or presence of different manipulations (e.g. heat stress, culture media components, maternal age). Coupled with immunofluorescence data, these experiments may also provide important evidence about synthesis and/or stability of specific mRNAs and thereby provide potential information about mechanisms associated with transcriptional and/or post-transcriptional regulation of mRNA abundance. Importantly, much less is known about changes in gene expression during oocyte growth. This is due to the technical difficulty of isolating pure cell populations. We propose that this method would enable tracking of not only oocyte-expressed candidate genes but also candidate genes expressed in granulosa and theca cells as follicles grow and develop toward the pre-ovulatory stage.

## Methods

### Mouse oocyte/embryo collection

All animal procedures were reviewed and approved by the Institutional Animal Care and Use Committee at the University of Nebraska-Lincoln and all methods were performed in accordance with relevant guidelines and regulations. For this specific study, CD-1 outbred mice had ad libitum access to water and normal rodent chow (Harlan Teklad, T.2918.15); they were maintained on a 12:12 dark: light cycle. At 6–8 weeks of age female mice were stimulated with 5 IU equine chorionic gonadotropin (eCG) and 5IU human CG (hCG) as described^39^. Cumulus-oocyte-complexes (COCs) containing germinal vesicle (GV) stage oocytes were collected 44 hours after eCG by puncturing antral follicles on the ovarian surface with a 27-gauge needle, while unfertilized MII oocytes were collected from the oviduct ampulla 16 hours post-hCG. To collect 1- and 2-cell embryos, eCG/hCG-stimulated females were placed with intact males of proven fertility overnight. Presumptive one-cell embryos were collected from the oviduct ampulla 16 hours after hCG stimulation. Two-cell embryos were flushed from the oviduct 1.5 days after mating.

### Oocyte/embryo fixation and single molecule RNA florescent *in situ* hybridization assay (RNA-FISH)

Freshly isolated cumulus-oocyte complexes, MII oocytes and pre-implantation embryos collected from at least 3 CD-1 mice per developmental stage were fixed in 100 μL drop of 4% paraformaldehyde with 0.1% polyvinylpyrrolidone (PVP) for 20 min, washed through 3 drops of wash buffer (0.1% Triton X-100 and 0.1% PVP in 1 × PBS) and permeablized (1% Triton X-100 in 1 × PBS) for 30 min. After permeablization oocytes and embryos were placed in wash buffer for 10 min prior to hybridization steps. Each hybridization step was performed in solutions within one well of an Agtech 6-well Solution dish (D18, Agtech, Manhattan, KS).

#### Affymetrix ViewRNA Cell Assay Hybridizations

Oocytes and embryos were subjected to limited protease digestion using 80 μl of QS diluted 8000-fold in 1 × PBS. Following the 5 min protease treatment oocytes and embryos were transferred to 100 μL wash buffer for 10 min. Permeablized and protease-treated oocytes and embryos were subsequently transferred to 80 μL of probe-containing solution (proprietary ViewRNA ISH probe sets for murine *Gdf9*, *Pou5f1*, or *Nanog* (see Table 1) diluted 1:100 in QF diluent) and incubated for 3 hours at 40 °C. For the studies presented, oocytes and embryos were co-hybridized with probes for *Pou5f1* and *Nanog* while single hybridizations were performed using *Gdf9*. Negative control cells were hybridized in QF diluent with no probe set added. Due to the specific gravity of the probe containing solution, oocytes and embryos tend to float and become transparent. It was crucial that oocytes and embryos were fully submerged during the entire 3-hour incubation. Following probe hybridization, oocytes and embryos were washed and then subjected to sequential hybridization with pre-amplifier DNA, amplifier DNA and fluorophore (*Gdf9*, LP1-550; *Pou5f1*, LP6-650; and *Nanog*, LP4-488). Each of the proprietary hybridization DNAs/label were diluted 25-fold in the provided diluent. Hybridizations with pre-amplifier, amplifier, and fluorophore were performed at 40 °C for 30 min each.

#### Advanced Cell Diagnostics RNAscope Hybridizations

Oocytes and embryos were subjected to limited protease digestion using 1 × Pretreat 4. After 5 minutes, oocytes and embryos were transferred to wash buffer for 10 min. Permeablized and protease-treated oocytes and embryos were subsequently transferred to 80 μL of probe-containing solution (proprietary RNAscope 3-plex Positive Control Probe Mm containing *M*. *musculus Ubc*, *Ppib*, *and Polr2ra* (see Table 1) combined as described by the manufacturer) and incubated for 2 hours at 40 °C. Alternatively, cells were hybridized with Negative Control probe containing *B*. *subtilis DapB* (see Table 1). Due to the specific gravity of the probe containing solution, oocytes and embryos tend to float and become transparent. It was crucial that oocytes and embryos were fully submerged during the entire 2-hour incubation. Following probe hybridization, oocytes and embryos were washed and then subjected to sequential hybridization with pre-amplifier and amplifier DNA (Amp1-FL, Amp 2-FL, and Amp 3-FL) and fluorophore (Amp4A ltB; *Ubc*-647 nm; *Ppib*-488 nm; *Polr2a*-550 nm; *DapB* -550 nm, 488 nm, and 647 nm). Hybridizations with pre-amplifier, amplifier, and fluorophore were performed at 40 °C for 15–30 min each as indicated by the manufacturer.

Following each hybridization step (regardless of the kit), oocytes or embryos were transferred through 3 wells of wash buffer. Oocytes and embryos were subsequently counterstained with 4′, 6-diamidino-2-phenylindole (DAPI) for 20 minutes prior to mounting on 25 × 75 mm microscope slides (Gold Seal®, 3039) in 12 uL ProLong® Gold Antifade Mountant reagent (P36934, ThermoFisher Scientific) and 25 × 25 mm coverslips (48368 084, VWR, Radnor, PA).

### Confocal Imaging and mRNA quantification in individual oocytes and embryos

Hybridized oocytes and embryos were imaged using a Nikon A1 “LSCM” on a Nikon-90 laser scanning confocal microscope at the University of Nebraska-Lincoln Center of Biotechnology Microscopy Core with image capturing assistance provided by Dr. Christian Elowsky. Sequential imaging was performed to avoid nonspecific fluorescence detection. Appropriate filter sets were applied based on the fluorophore used for each transcript (*Gdf9*, *Polr2a*, and *DapB* 550 nm; *Pou5f1*, *Ubc*, and *DapB* 647 nm; *Nanog*, *Ppib*, and *DapB* 488 nm) and Z-series sectioning performed. Individual Z-section images were visualized with NIS-Elements 4.4 image program and exported for compatibility with Image J. To count individual fluorescence signal indicative of a single mRNA molecule, images for each section were maximum-projected and stitched together in Image J Fiji using the Grid/Collection plug-in^27^ to form a composite image (1.45 S, Wayne Rasband, NIH, USA). Individual spots of fluorescence were located and counted in each composite image using the software program Localize which was written in Interactive Data Language (ITT Visual Information Solutions)^40^. Specifically, the Localize program was run using each composite image and the default threshold of 10.0 photons and band pass threshold of 400 (Fig. 3A); the output of the program was the number of spots counted.

### Traditional RNA isolation, reverse transcription and droplet digital polymerase chain reaction (ddPCR) analysis of target transcripts

RNA was isolated from a pool of 15–20 oocytes or embryos collected from at least 2 CD-1 mice per developmental stage using Tri Reagent (Sigma-Aldrich) according to the manufacturer’s instructions. Isolated RNA was reverse-transcribed using MMLV-RT as previously described^41^. Complementary DNA (cDNA) from oocytes and embryos were diluted 2-fold prior to combination with 1XQX200 ddPCR Evagreen Supermix (BioRad Laboratories), which includes a proprietary SYBR green fluorescent dye and RNA polymerase, and 100 μM of gene specific primers (Table 1). In addition, primers were combined with either no template (PCR negative control) or synthetic-produced gBlocks Gene Fragments (10 pg/μL, Integrated DNA Technology, Table 1) which represented the PCR positive control. Each sample was emulsified by the QX200 Droplet Generator (BioRad) resulting in 20,000 droplets containing the Evagreen supermix (Bio-Rad), cDNA template with or without the targeted sequence, and target cDNA primers per reaction tube. Forty rounds of PCR amplification were performed using the C1000 Touch Thermal Cycler (Bio-Rad) and the number of droplets positive and negative for fluorescence for each sample was measured using the QX200 Droplet Reader (Bio-Rad). The copy number of target transcript per μl of cDNA was quantified using Quantisoft (Bio-Rad) and the number of target transcripts μ per oocyte or embryo calculated as follows:

1 $$\frac{({\rm{copy}}\,\#/{\rm{\mu }}{\rm{L}}\,{\rm{of}}\,\text{cDNA})\times {\rm{dilution}}\,{\rm{factor}}\times 25{\rm{\mu }}{\rm{L}}\,{\rm{cDNA}}}{\#\,{\rm{oocytes}}\,{\rm{or}}\,{\rm{embryos}}}$$

### Statistical Analyses

Comparison of *Gdf9*, *Pou5f1*, and *Nanog* mRNA abundance between *in vivo* produced GV-stage cumulus oocyte complexes, MII oocytes, 1-cell embryos, and 2-cell embryos was performed using GraphPad Prism 7.0 (GraphPad Software, La Jolla, CA). For each comparison, one-way ANOVA was performed followed by Tukey’s multiple comparison post-test. Data were presented as mean ± SEM. Differences in mRNA abundance between each oocyte or embryo stage were considered significant at *P* < 0.05.
